# Multi-institutional prognostic modeling of survival outcomes in NSCLC patients treated with first-line immunotherapy using radiomics

**DOI:** 10.1186/s12967-024-04854-z

**Published:** 2024-01-10

**Authors:** Sevinj Yolchuyeva, Leyla Ebrahimpour, Marion Tonneau, Fabien Lamaze, Michele Orain, François Coulombe, Julie Malo, Wiam Belkaid, Bertrand Routy, Philippe Joubert, Venkata SK. Manem

**Affiliations:** 1 Department of Mathematics and Computer Science, Université du Québec à Trois Rivières, Trois-Rivières, Canada; 2 Quebec Heart & Lung Institute Research Center, Québec , Canada; 3 Centre de Recherche du Centre Hospitalier Universitaire de Montréal, Montreal, Canada; 4 Department of Molecular Biology, Medical Biochemistry and Pathology, Laval University, Québec, Canada; 5 Université de médecine de Lille, Lille, France; 6 Centre de Recherche du CHU de Québec, Université Laval, Québec, QC Canada; 7 Department of Physics, Laval University, Québec, Canada

## Abstract

**Background:**

Immune checkpoint inhibitors (ICIs) have emerged as one of the most promising first-line therapeutics in the management of non-small cell lung cancer (NSCLC). However, only a subset of these patients responds to ICIs, highlighting the clinical need to develop better predictive and prognostic biomarkers. This study will leverage pre-treatment imaging profiles to develop survival risk models for NSCLC patients treated with first-line immunotherapy.

**Methods:**

Advanced NSCLC patients (*n* = 149) were retrospectively identified from two institutions who were treated with first-line ICIs. Radiomics features extracted from pretreatment imaging scans were used to build the predictive models for progression-free survival (PFS) and overall survival (OS). A compendium of five feature selection methods and seven machine learning approaches were utilized to build the survival risk models. The concordance index (C-index) was used to evaluate model performance.

**Results:**

From our results, we found several combinations of machine learning algorithms and feature selection methods to achieve similar performance. K-nearest neighbourhood (KNN) with ReliefF (RL) feature selection was the best-performing model to predict PFS (C-index = 0.61 and 0.604 in discovery and validation cohorts), while XGBoost with Mutual Information (MI) feature selection was the best-performing model for OS (C-index = 0.7 and 0.655 in discovery and validation cohorts).

**Conclusion:**

The results of this study highlight the importance of implementing an appropriate feature selection method coupled with a machine learning strategy to develop robust survival models. With further validation of these models on external cohorts when available, this can have the potential to improve clinical decisions by systematically analyzing routine medical images.

## Introduction

Systemic treatments have been the mainstay for treating advanced non-small cell lung cancer (NSCLC), however, the benefit from these therapeutic regimens has reached a plateau [1]. They are also associated with significant toxicities along with limited overall and progression-free survival. Over the last few years, the use of immune checkpoint inhibitors (ICIs) has drastically changed clinical care and therapeutic paradigms in advanced and metastatic NSCLC patients [2]. With numerous clinical trials indicating prolonged survival with ICIs compared to platinum-based treatments, the application of ICIs has made inroads to the first-line setting for treating advanced NSCLC patients as well [3].

In recent years, to improve the outcomes of NSCLC patients, there has been an increasing number of combinations of ICIs with other immunotherapeutic agents, radiotherapy, targeted compounds, and chemotherapies [4, 5]. There are numerous clinical trials that are currently ongoing for implementing first-line therapies in lung cancer - CheckMate 9LA (chemo with or without nivolumab and ipilimumab) [6], B-FAST (single or a combination of targeted compounds and ICIs) [7], and KEYNOTE (pembrolizumab with ipilimumab) [8]. As more patients are treated with ICIs as monotherapy or in a combination setting, there will be failures accompanying them too [3]. Therefore, future efforts must be directed towards developing strategies to overcome disease progression or therapy resistance to improve patient-specific outcomes. This involves understanding the biological mechanisms driving disease progression and resistance during immunotherapy as well as developing novel predictive and prognostic biomarkers to better select patients who can be treated with ICIs. Eventually, this will help us to improve the long-term patient-specific outcomes, after receiving first-line ICIs [9]. More importantly, the decreased survival benefits in advanced NSCLC patients may represent that a subset of patients either do not respond to ICIs and are amenable to other therapies. Given that NSCLC exhibits heterogeneity in tumor biology, clinical presentation, and treatment response [10], it becomes crucial to select an optimal therapeutic strategy for NSCLC patients treated with first-line ICIs. Therefore, there is a dire need to build prognostic tools that can guide clinical decision making. Moreover, developing a robust biomarker can potentially spare these patients from toxicities induced by the unnecessary administration of ICIs.

Recent advances in computational imaging approaches, radiomics, the process of extracting descriptors from routine medical images by mathematical functions, have led to a large set of quantitative imaging features to study biological and clinical endpoints [11, 12]. This is a non-invasive technique and the human-interpretable radiomics features can be extracted from routine radiological images (such as CT-scan and MRI scan) and quantifies tumor characteristics in a high-throughput manner. A number of imaging-driven models have shown promising results in oncological applications, particularly, in building predictive and prognostic biomarkers in a variety of malignancies, including NSLC, melanoma, and other types of cancers. In a study led by Zerunian et al., the authors built a CT-based imaging model to predict OS and PFS in 21 patients presented with advanced NSCLC who were administered with first-line pembrolizumab [13]. One of the drawbacks of this work is that their model was validated on a small dataset consisting of 21 patients, and larger cohorts were needed to confirm the results to translate them to a clinical setting. Braghetto et al. compared imaging signatures derived from engineered features and deep learning-based features to predict the 2-year OS in NSCLC patients leveraging the publicly available lung cancer dataset [14]. A recent comparative study by Li et al. the authors used 3 machine learning models along with EHR data to build predictive models for clinical endpoints using data from a single institution [15]. While there have been several promising prognostic modeling studies leveraging radiomics data, the adoption of radiomics-based prognostic models in clinical workflows is still limited [16, 17]. There are several factors that affected adopting these models in clinical settings, which include the lack of a clear predictive feature selection and learning strategy, relatively small sample size and single-institution datasets lacking external validation as well as generalizability. Moreover, none of these studies have attempted to build survival predictive models from a multicenter perspective to predict PFS and OS in NSCLC patients treated with first-line immune checkpoint inhibitors - Nivolumab and Pembrolizumab.

Nevertheless, prognostic model development is complex, as no modeling approach is better than other strategies, and validation of these models requires diverse cohorts to illustrate that the developed models are applicable to several other external datasets. With this premise, in this study, we performed a systematic exploration of a compendium of feature selection and machine learning strategies to develop prognostic models for OS and PFS, leveraging multi-institutional pre-treatment CT-scan data. To achieve this, we used two independent cohorts from different institutions to build the biomarkers, which will make them more generalizable for a clinical setting. To the best of our knowledge, no studies in the literature have done this systematic comparison on two independent cohorts of patients treated with first-line ICIs. Overall, this work will help us to identify an appropriate configuration of feature selection and machine learning methods to develop clinically robust survival models for future work that leverages radiomics data. This understanding, combined with the generation of numerous imaging datasets across centers in the future, will bring closer the era of translating AI-driven tools to the clinic.

## Materials and methods

### Study population

This retrospective study includes 149 patients who were presented with advanced NSCLC and treated between 2015 and 2021 with first-line immune checkpoint inhibitors. The cohorts were obtained from two research institutions: Institut Universitaire de Cardiologie et de Pneumologie de Québec (Quebec Heart and Lung Institute, IUCPQ) and Centre Hospitalier Universitaire de Montréal (CHUM). The samples from the IUCPQ came from the Quebec Respiratory Health Network Tissue Bank (https://rsr-qc.ca/biobanque/). The study was approved by the Institutional Review Boards at the two academic institutions where the data was collected (MP-10-2020-3397 / CÉR CHUM: 19.397). All patients with advanced NSCLC and who were treated with first-line ICIs and had a pre-ICI CT scan were eligible for retrospective review. The Response Evaluation Criteria in Solid Tumors (RECIST) version 1.1 criteria were utilized to evaluate tumor response. The radiologist’s qualitative assessment determined the progression. Every patient was monitored until either their death or the date of censoring, which was set to coincide with the last time the individual was known to be alive, which was in January 2022. As treatment options, radiation and chemotherapy were used. In both cohorts, chemotherapy was given before ICIs. In the IUCPQ cohort, 46 patients received radiation therapy prior to immunotherapy with ICIs, while 8 patients underwent the reverse sequence. For the CHUM cohort, this information was unavailable. There are potential variations between the IUCPQ and CHUM cohorts in terms of the CT scanner type, slice thickness, kernel, etc. For the CHUM cohort, the CT images of the patients were acquired using scanners from four different companies - Siemens with slice thickness {0.75 mm, 1 mm, 2 mm, 5 mm}, GE Medical Systems with slice thickness {2.5 mm, 5 mm}, Philips with slice thickness {2 mm, 5 mm} and Toshiba devices with slice thickness {1 mm, 2 mm}. While for the IUCPQ cohort, the CT images of the patients were acquired using scanners from three different companies - Siemens with slice thickness {2 mm}, GE Medical Systems with slice thickness {1.25 mm, 2.5 mm}, and Philips devices with slice thickness {2 mm, 3 mm}. A total of 149 people made up the dataset put together by the two centers, including 95 patients from the discovery cohort (CHUM) and 54 patients from the in-house validation cohort (IUCPQ).

### Clinical endpoints

Progression-free survival (PFS), is defined as the amount of time that passes between the beginning of treatment and the onset of disease progression. The date of illness progression, date of death from any cause, or date of last follow-up (censored) is used to calculate survival time. CT scans, MRIs, and PET-CT scans are frequently used to confirm the development of the disease. The overall survival, or OS rate is calculated from the patient’s date of diagnosis until the date of death from any cause or the date of censoring, whichever comes first. OS and PFS were viewed as regression-based tasks.

### Radiomics features

The radiomics features were extracted and classified into four categories, including tumor intensity-, shape-, texture-, and wavelet-based features, by utilizing PyRadiomics (v 3.0.1), the open-source Python library [18, 19]. First order statistical features based on a histogram of all voxel intensity values are intensity-based features that describe the properties of tumor intensity. The shape of the tumor is characterized by features like its sphericity or compactness. Variations in texture within the tumor volume are characterized by textural features. To do this, voxels with similar appearance were clustered using either a gray-level co-occurrence matrix, a run-length gray-level matrix, or a gray-level size-zone matrix. All scans’ slice thicknesses were interpolated to voxel sizes of 1 × 1 × 1mm^3^, and features were computed in 3D. The image was transformed using a Laplace of Gaussian operator, and then wavelet-based features were computed using the intensity and texture information. These features were utilized as input for building the predictive models. The Pyradiomics radiomics platform was used to extract the radiomics characteristics from segmented tumor regions of pre-treatment CT scans from two separate cohorts for developing survival risk models. The CHUM cohort was used as the discovery cohort, whereas the IUCPQ cohort was used as the validation dataset to assess the prediction performance for PFS and OS tasks.

### Overview of modeling approaches

When working with high-dimensional datasets to build models, where the number of features is considerable in comparison to the number of samples, feature selection is especially crucial [20]. In such cases, not all features might contribute equally to a model’s predictive power, and some might even introduce noise or redundant information. To create the predictive models for OS and PFS in this study, we used a two-step feature selection process. To minimize the dimensionality of the feature space, we initially used the least absolute shrinkage selection operator (LASSO) method. Following that, five feature selection approaches were used to develop the radiomics-based model, which are called as analysis of variance (ANOVA)-F-test (AFT), Mutual Information (MI) [21], ReliefF (RL), Multisurf (MSF), and Surf (SF) [22–25]. The regressor was constructed by first selecting the most important characteristics using the measured estimates acquired for each feature using the following approaches. Various feature selection methods used in the study are described below:

i) AFT: It is a statistical test designed to investigate the differences between numerical and categorical sets of data. The F-statistic is used to rank each of the features in the data, and the features with the highest scores are chosen as the optimal set.
ii) MI: It is a method used to identify the most informative features in a dataset based on their mutual information with the target variable [21, 26]. Mutual information measures the statistical dependence between two variables by quantifying the amount of information obtained about one variable through the other. If the MI value is near to zero, the association between the feature and the target is weak.
iii) RL, MSF, and SF: The Relief algorithm is a popular and effective feature selection method in machine learning. It was originally proposed by Kira and Rendell in 1992 [27, 28]. It is among the most widely used non-myopic algorithms for feature ranking, where each feature is assigned a real-valued score, offering insights into its importance [28]. Extensions and variations of the Relief algorithm have been proposed to address specific challenges or enhance its performance. These variations include ReliefF, Surf, Multisurf, etc., which incorporate additional considerations or modifications to the original algorithm [22]. Urbanowicz et al., created scikit-rebate, an open-source Python software package that includes RL and its modifications [22]. Specifically, the package added support for binary classification, multi-class classification, regression, discrete, continuous, and mixed feature types, and introduced a new core RBA called Multisurf (MSF) and Surf (SF).

**Fig. 1:**
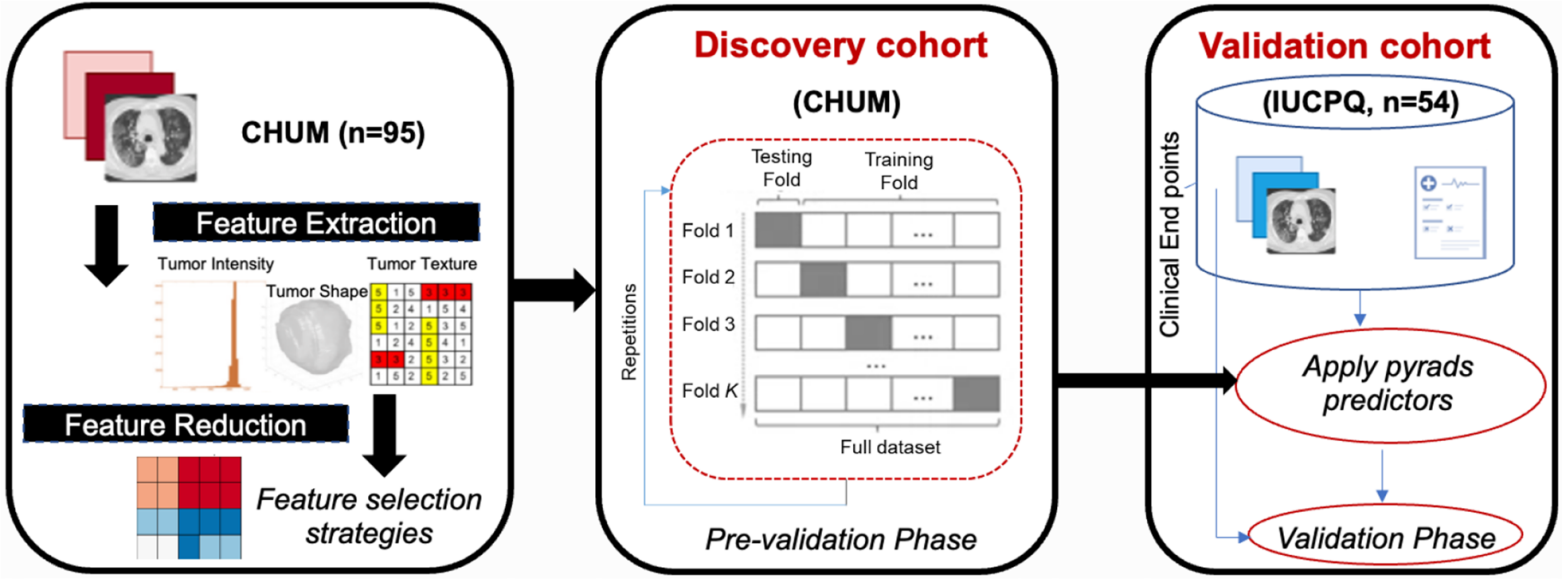
The workflow of radiomics analysis used in this study

In our study, both PFS and OS were considered as regression tasks. To build the survival endpoints, we utilized seven different machine learning models: Adaptive boosting (AdaBoost), Decision Tree (DT), Random Forest (RF), Linear regression (LR), K-nearest neighbourhood (KNN), Gradient Boosting (GBoost), eXtreme Gradient Boosting (XGBoost). All the regression-based approaches were implemented using the Sklearn package, which makes it easy to utilize many machine-learning algorithms in Python. The analysis pipeline is illustrated in Fig. 1.

The following procedure was carried out to create the radiomic-based predictive models. Firstly, we performed data standardization, i.e., all features in both cohorts were transformed to have a mean of zero and a standard deviation of one. Feature selection and classification training were done using the discovery cohort, CHUM, and the performance was evaluated in the independent validation cohort, IUCPQ. Firstly, to decrease the number of features, LASSO regression was applied to the entire training cohort. For this purpose, we used grid-search with 5-fold cross-validation to determine the best alpha for LASSO. After training LASSO on the entire CHUM cohort, we eliminated features with zero coefficients. After removing features with zero coefficients from the 851 radiomics features, the remaining features were 41 for OS and 25 for PFS. The rest of the procedure can be split into two phases: the pre-validation phase and the validation phase.

We started by evaluating the performance of each model in the pre-validation phase, which was conducted in the discovery set (CHUM). This phase consisted of a 5-fold cross-validation for each of the models mentioned above. Each of the feature selection methods was combined with each of the seven machine learning models, and the features were incremented to find a subgroup of features with the best performance in terms of C-index for OS and PFS iteratively. In other words, for each of the feature selection techniques, we iteratively picked features from 3 until the number of rest features (using the features that remained after applying LASSO) under each cross-fold. The approach that yielded the greatest score for OS and PFS was determined to be the best one for selecting features, and that method’s best feature number was identified. The approach that yielded the greatest score for OS and PFS was determined to be the best one for selecting features, and that method’s best feature number was identified. In the validation phase, we trained each of the models on the CHUM dataset with best features, then evaluated them on the IUCPQ dataset with the best features. Finally, we computed the C-index for OS and PFS for the validation phase (similar to our previous works [17, 29]). In addition, the hyperparameter tuning of the classifiers was carried out through the process of cross-validation with the help of the GridSearchCV class provided by scikit-learn. The scikit-learn module, version 1.0.2, was used to implement the feature selection and classification techniques. Python version 3.9.13 was used for the implementation.

## Results

### Patient characteristics

The clinical characteristics of all NSCLC patients treated with first-line ICIs in discovery and discovery cohorts are summarized in Table 1. The continuous values were presented as mean ± standard deviation (SD) and the categorical data as both counts and percentages.

**Table 1:** Clinical characteristics of discovery and validation cohorts

Characteristics	CHUM (discovery)	IUCPQ (Validation)
# Of samples	95	54
Age (mean)	67.9 ± 8.1	67.1 ± 7.3
Gender, n (%)		
Female	51 (54%)	32 (59%)
Male	41 (46%)	22 (41%)
Smoking status, n (%)		
Former	68 (73.9%)	34 (63%)
Current	22 (23.9%)	16 (29.6%)
Never	2 (2.2%)	4 (7.4%)
ECOG status, n (%)		
0	37 (39%)	20 (37.1%)
1	41 (43.1%)	32 (60%)
2	14 (14.7%)	1 (1.9%)
3	3 (3.2%)	1 (1.9%)
Histology, n (%)		
Adenocarcinoma	77 (81%)	41 (76%)
Squamous	14 (15%)	8 (14.8%)
Other	4 (4%)	5 (9.2%)
EGFR status, n (%)		
Absent	75 (79%)	47 (87%)
Present	3 (3.1%)	2 (3.7%)
NT	17 (17.9%)	5 (9.3%)
PFS, n (%)		
6 months	51 (54%)	21 (38.9%)
6 months	44 (46%)	33 (61.1%)
OS, n (%)		
6 months	78 (82%)	45 (83.3%)
6 months	17 (18%)	9 (16.7%)

The discovery cohort CHUM consists of 95 patients with an average age of 67.9 years (± 8.1). Within the smoking category, 73.9% of the patients were former smokers and 23.9% of them still smoke. The validation cohort, IUCPQ, consists of 54 patients with an average age of 67.1 years (± 7.3). Among these patients, 63% of them were former smokers and 29.6% still smoke. The ECOG performance status is measured on a scale from 0 to 5, where 0 denotes a person who is entirely active and in the same physical condition as before the disease, and 5 indicates a person’s mortality. The majority of patients in both cohorts are labeled with an ECOG performance status of 1. In the discovery and validation cohorts, PFS varied between 0.20 and 49.1 months and 0.13–57.7 months, respectively. The OS values ranged between 0.30 and 49.1 months in the discovery cohort and 0.13–47.4 months in the validation cohort.

### Predictive performance of models

**Fig. 2:**
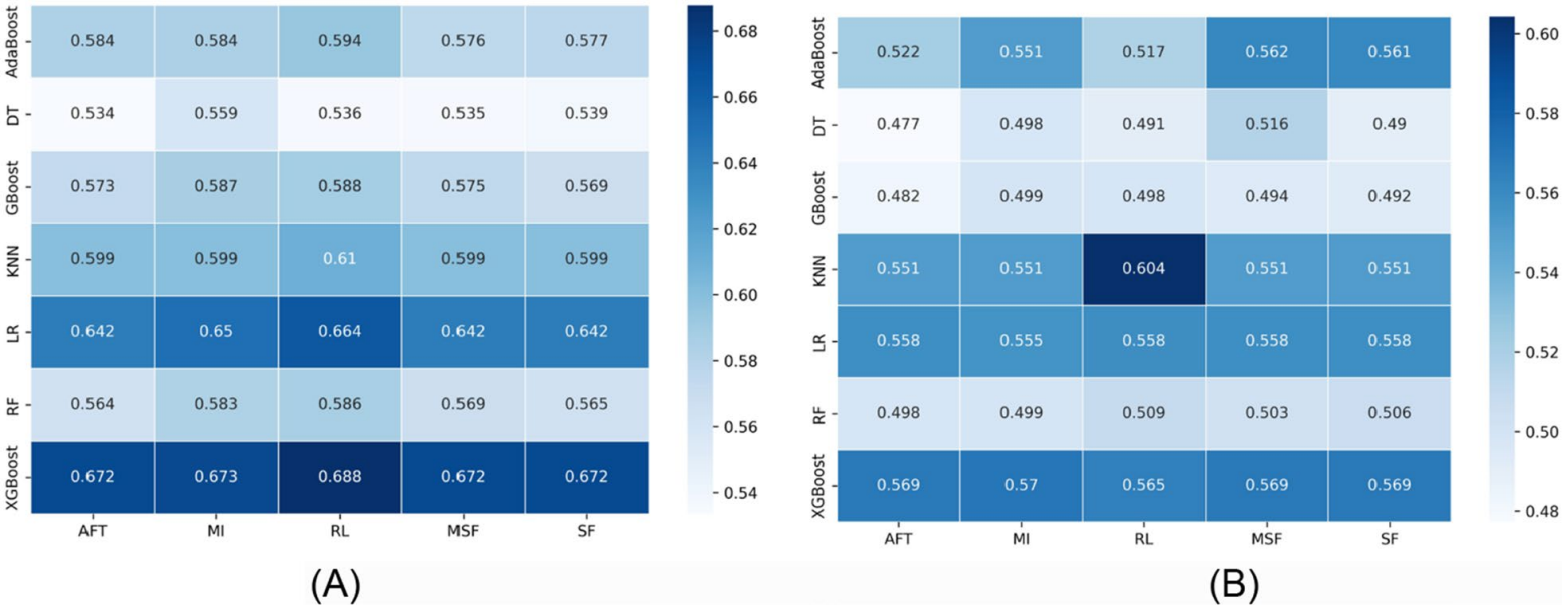
Heatmaps illustrating the performance of each machine learning algorithm (rows) with each feature selection method (columns) for the PFS task. **A** C-index in the cross-validation phase, and **B** C-index in the validation phase

**Fig. 3:**
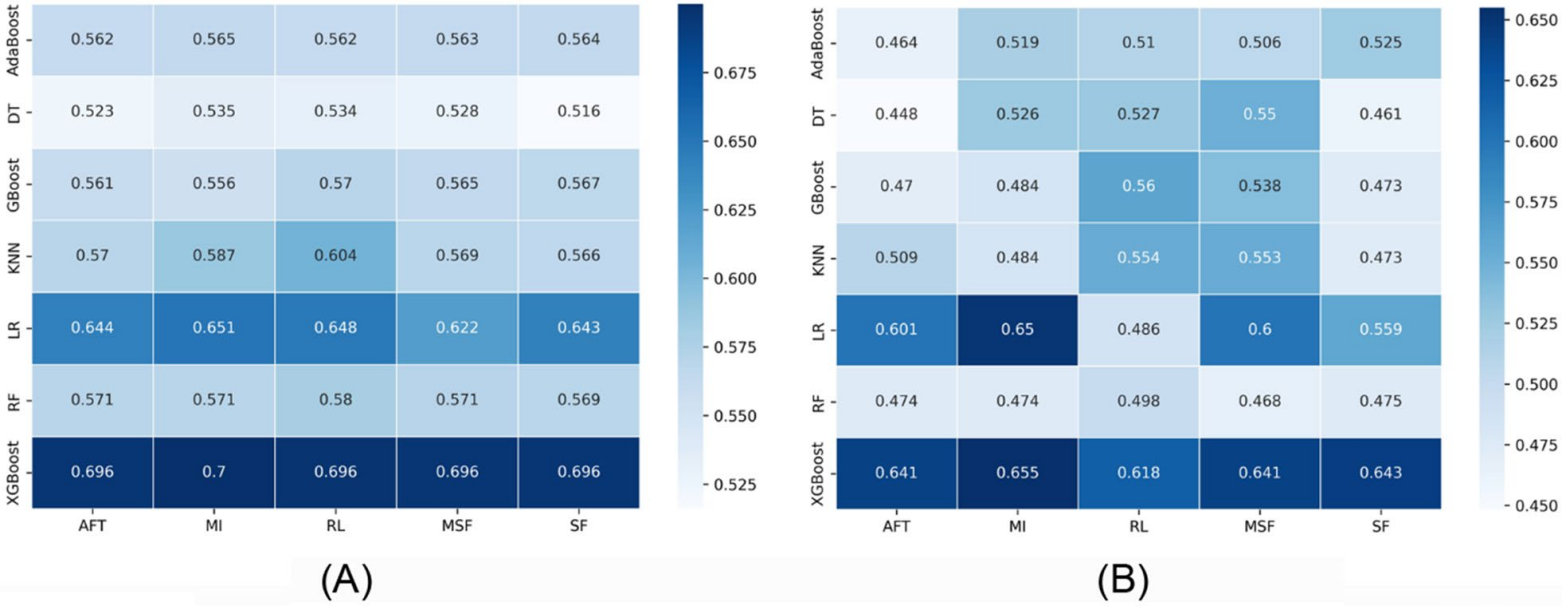
Heatmaps illustrating the performance of each machine learning algorithm (rows) with each feature selection method (columns) for the OS task. **A** C-index in the cross-validation phase, and **B** C-index in the validation phase

The C-index was used to analyze the prediction performance of various feature selection and machine learning methods. Figures 2 and 3 show the performance of feature selection approaches (in columns) and machine learning methods (in rows) for PFS and OS, respectively. There were five C-index values for each approach, which corresponded to the five separate feature selection methods. Figure 2 A and B show the C-index scores for the PFS discovery and validation cohorts, respectively.

In the discovery cohort, XGBoost showed similar performance with 5 different feature selection methods (C-index = 0.672–0.688); however, the RL feature selection method with KNN showed the best performance in the validation cohort (C-index = 0.604). Although the combination of LR with various feature selection methods shows better performance in the discovery cohort, the C-index scores are under 0.60 in the validation cohort. The DT, RF, and GBoost models displayed the worst performance (C-index = 0.47–0.51) in the validation cohort.

Figure 3 A and B present the C-index for OS in the discovery and validation cohorts, respectively. The performance of the LR and XGBoost models with all feature selection models was found to be better than the others in the discovery cohort, with the C-index score higher than 0.60. With the MI feature selection method, XGboost and LR demonstrated the highest C-index in the validation cohort (C-index = 0.65–0.655). In the validation dataset, the RF model with any feature selection method displayed the worst performance (C-index = 0.468–0.498).

### Median performance of learning methods

**Fig. 4:**
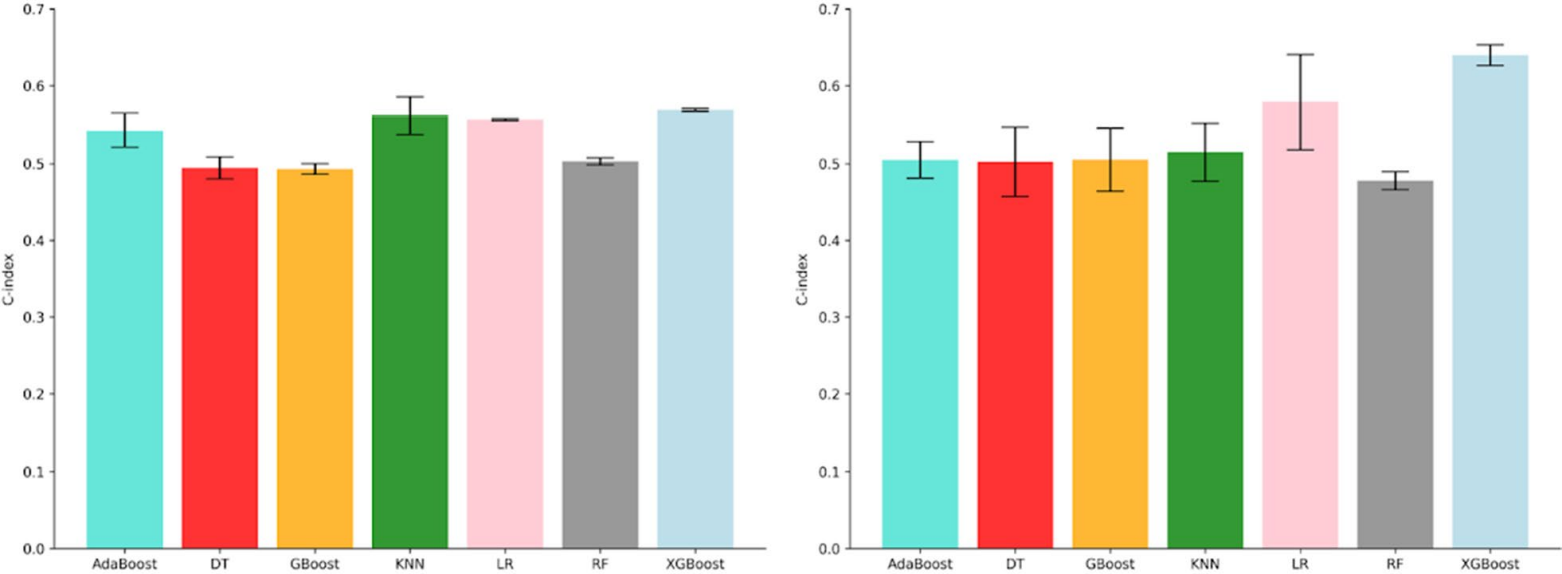
Median performance of machine learning methods to predict PFS and OS on the validation dataset

We computed the median performance of the classifiers across all feature selection methods for OS and PFS, which is displayed in Fig. 4. For OS, the highest median performances were obtained by XGBoost (C-index: 0.64 ± 0.013), followed by AdaBoost (C-index: 0.582 ± 0.034). For PFS, the highest median performances were obtained by the XGBoost (C-index: 0.57 ± 0.001, median ± std), followed by the KNN (C-index: 0.56 ± 0.023, median ± std). The lowest model performance was achieved by RF and DT to predict both PFS and OS. Most importantly, we found several combinations of feature selection methods and machine learning algorithms to achieve a similar median score for both tasks.

## Discussion

For several decades, the mainstay of treating advanced or metastatic non-small-cell lung cancer (NSCLC) consisted of platinum-based chemotherapeutic agents and targeted therapies, which resulted in moderate survival outcomes [1]. In this therapeutic landscape, the recent development of immune checkpoint inhibitors have revolutionized the treatment of NSCLC patients. In less than a decade, ICIs have significantly improved the survival outcomes of patients presented with NSCLC in a first-line setting [9]. However, only a subset of patients get benefitted from these therapies. Important questions remain on how to better select NSCLC patients and optimize the therapeutic administration of these expensive compounds. With the ever increasing arsenal of OMICS data, there have been several efforts to develop prognostic and predictive biomarkers using gene expression profiles, however, none of these OMICS-based signatures have been translated to a clinical setting, which requires a rigorous development and validation as well as adhere to some regulatory processes [30].

This has propelled research towards building imaging-based prognostic and predictive biomarkers utilizing routine medical images [17]. Recent studies have leveraged CT-scans of NSCLC patients treated with ICIs and developed imaging biomarkers to predict immunotherapy response and survival outcomes [17]. To better identify the subset of patients who will be benefited from ICIs, imaging-based biomarkers are being built to predict patient-specific outcomes (for both response and survival) among NSCLC cohorts. It is unclear from the published retrospective studies whether the developed models can translate to a clinical workflow. The lack of a clear feature selection and learning methodology, small sample sizes of patients, lacking external validation and generalizability have impeded the clinical translation of these imaging signatures. Furthermore, it is not unknown from the literature to identify which configuration of feature selection strategy combined with a machine learning algorithm will lead to the highest accuracy in a multi-institutional setting. A systematic comparison of a spectrum of feature selection and machine learning strategies has not been attempted before in the context of a multicentric setting for NSCLC patients treated with first-line ICIs. Building and validation of radiomics models on larger cohorts across hospitals will ensure us to move towards clinical translatability.

With this premise, we investigated the utility of several feature selection strategies combined with machine learning algorithms to predict survival outcomes (PFS and OS), by leveraging handcrafted features from CT-scan data. The strengths of our study include the cohorts of patients evaluated at two institutions (*n* = 149) in terms of diversity of patient populations, data heterogeneity across centers, and the use of two clinical endpoints, OS and PFS. We employed five feature selection methods (i.e., AFT, MI, RL, SF, and MSF) and seven machine learning algorithms (i.e., AdaBoost, DT, GBoost, KNN, LR, RF, and XGBoost) to build the prognostic models. Both PFS and OS were considered as regression-based tasks. To predict PFS, we found the performance of LR and XGBoost to have an almost similar C-index in the discovery cohort. In the validation cohort, the RL feature selection approach with KNN was found to have the highest performance (C-index = 0.604) among all combinations of other machine learning models and feature selection methods. For predicting the OS, the performance of the LR and XGBoost models with all feature selection models was found to be better than the other configurations in the discovery cohort, with the C-index score higher than 0.60. While in the validation cohort, XGBoost and LR with the MI feature selection method demonstrated the highest C-index (C-index = 0.65–0.655). Moreover, we also observed that DT, RF, and GBoost models with some feature selection methods showed the worst performance (C-index = 0.47–0.51) in the validation cohort for both PFS and OS.

In a recent study by Li et al. [15], the authors have developed predictive models for OS and PFS using an NSCLC cohort treated with first-line ICIs from a single center by applying three machine learning approaches. They obtained concordance indices of 0.67 and 0.61 for OS and PFS on the validation tests (that was held out within the same center). The model performance was achieved by incorporating the clinical parameters such as ECOG status and PD-L1 expression. While in our study, we carried a more comprehensive exploration of a compendium of feature selection and machine learning approaches for building the clinical endpoints. From our findings, we obtained almost similar concordance indices of 0.66 and 0.61 to predict OS and PFS, respectively, on the validation dataset, without adding any clinical variables. More importantly, we developed and validated our models in a multi-institutional setting which is more likely to be robust and generalizable, with further validation on multiple centers.

Our study has several potential limitations. Firstly, the heterogeneity of scanners across centers along with the impact of bin-level discretization on the radiomics feature extraction were not accounted for while building the predictive models. The validation of radiomics models was done on the retrospective cohort, and not on the prospective dataset. Finally, the potential biases of the study design across the two institutions might have also impacted the model accuracy. By incorporating image harmonization methods [31] to correct for the variations in acquisition parameters, we believe the performance of the models can be potentially improved, which we are exploring in our future study. In summary, we investigated a landscape of feature selection and machine learning approaches for building models to increase their clinical translatability and generalizability. Through this study, we have identified significant imaging predictors of survival endpoints utilizing a landscape of feature selection and modeling strategies, which also facilitates model interpretation. As such, this study highlights the importance of implementing an appropriate feature selection method combined with a machine learning approach to develop clinically usable prognostic models for patients treated with ICIs in a first-line setting. This will pave the way to integrate AI-driven tools in clinical workflows, thereby improving the therapeutic outcomes of NSCLC patients, by systematically analyzing routine medical images.

## Data Availability

Data presented in this study are not publicly available at this time but may be obtained from the corresponding author, Venkata Manem upon reasonable request.
